# “To Be or Not to Be” a Conscientious Objector to Voluntary Abortion: An Italian Web-Survey of Healthcare Workers

**DOI:** 10.3390/medicina60121984

**Published:** 2024-12-02

**Authors:** Carmen Imma Aquino, Libera Troìa, Maurizio Guida, Daniela Surico

**Affiliations:** 1 Department of Translational Medicine, University of Piemonte Orientale, Gynecology and Obstetrics, ‘Maggiore della Carità’ Hospital, 28100 Novara, Italy; c.immaquino@gmail.com (C.I.A.); libera.troia@uniupo.it (L.T.); 2 Department of Neurosciences and Reproductive Sciences, University of Naples Federico II, 80131 Naples, Italy; profmaurizioguida@gmail.com

**Keywords:** abortion, interruption of pregnancy, family planning

## Abstract

*Background and Objectives*: Conscientious objection to voluntary abortion remains a hot debate topic. This could affect the accessibility to pregnancy termination. Our aim is to evaluate the possible aspects related to an operators’ choice about objection for voluntary abortion, such as the following: the abolition of the time limit, the instruction of a multi-collegiate commission, the introduction of pharmacological rather than surgical procedures, the fetal/maternal illness and the case of sexual violence. *Materials and Methods*: This is an observational, descriptive study that involves a cohort of Italian healthcare workers who answered a web-survey. *Results*: Of the total 352 respondents, only 20.8% affirmed to be objectors versus 79.2% of non-objectors. For the objectors, 72.2% declared that they would not change status in case of pharmacological abortion; 79.7% would not suspend their choice for interruption in the second trimester; 63.3% would suspend the objection with a multi-collegiate commission, and 69.0% would discontinue their objection in the case of sexual violence. 72.0% of the total participants declared that the abolition of the time limit could have a resecuring impact on women’s choice. *Conclusions*: Most operators declared that the abolition of the time limit could have beneficial effects. Among the objectors, the status would change especially with the introduction of a multi-collegiate commission, and in case of serious maternal/fetal illness and/or sexual violence.

## 1. Introduction

The term conscientious objection to abortion refers to the right of health workers as doctors, nurses, and midwives to refuse to participate in any way in the execution of the voluntary termination of pregnancy (TOP) because they deem it unfair and contrary to their ethical and moral values and principles, except if the objector’s action is indispensable to save the woman’s life, such as in cases of imminent danger [1].

Voluntary TOP was legalized in Italy with Law no. 194/78 (“Rules for the social protection of maternity and on voluntary termination of pregnancy”). The situation abroad is a little more varied [1]. In Italy, there is a greater diffusion of conscientious objectors than in other nations; this occurs for various reasons (e.g., cultural, religious) and may cause possible difficulties in guaranteeing the abortion service [2]. Contrary to what happens in other countries, abortions in Italy can only be performed by gynecologists, and never by general practitioners, even when the abortion could be obtained pharmaceutically using RU486 (Mifepristone) [2]. In 2022, Italian objectors among healthcare professionals comprised the following: 64.6% of gynecologists, 44.6% of anesthetists and 36.2% of other healthcare personnel. Access to voluntary abortion could also be more complex in some specific areas (i.e., Southern Italy) [2].

The numbers of TOP in Italy remain among the lowest internationally; the abortion rate (number of TOP per 1000 women residents aged 15–49 years) was equal to 5.4 per 1000 in 2020 (−6.7% compared to 2019) [3].

The situation is different in Europe, where the percentages of objector personnel are as follows: 3.0% in France, 6.0% in Norway and Germany, and 10.0% in England. In Sweden and Finland, conscientious objection is not contemplated. The situation in Austria and Croatia is more complex and like that of the Italian legislation [4,5]. In Italy, 56.0% of surgery abortions were carried out within 8 weeks of gestation (compared to 53.3% in 2019). The percentage of TOP within the 8 weeks has increased in recent years (in 2012, it was 41.8%), probably due to the use of Mifepristone and prostaglandins (pharmacological abortion) preferred in earlier gestational age. Furthermore, there is a slight tendency towards an increase in the percentage of TOP beyond 12 weeks of gestation [3].

It is important to specify the distinction between voluntary TOP and therapeutic termination of pregnancy. Voluntary TOP is a procedure that a woman in Italy can request within 90 days of conception for health, economic, family and social reasons. Therapeutic termination of pregnancy can be requested by the woman within 180 days of conception due to danger to her life or to serious danger to her physical or mental health. Methods could be surgery or drug therapy, with more complications at advanced gestational weeks. Voluntary abortion beyond 180 days can only be requested in countries such as England or France; one of the most used procedures is the administration of potassium chloride to the fetus, causing fetal cardiac arrest [1,6].

Before 12 gestational weeks, there are mostly unwanted pregnancies, preventable by promoting responsible procreation; abortion after 12 weeks of pregnancy is frequently associated with unfavorable fetal outcomes or maternal pathologies. Although the former tend to reduce over time due to responsible contraception use, the latter is increasing with the greater implementation and improvements in prenatal diagnosis and the increased maternal age [3].

These situations are stressful not only for patients but also for the healthcare workers involved. When considering their psychological involvement as well, it may be that the objectors’ attitude is not the same for each clinical situation, for example, in the case of TOP in the first (up to 12 gestational weeks) or second trimester (allowed in Italy up to 22 gestational weeks), or in making a distinction according to the conditions of the mother and/or embryo/fetus [7,8]. The fact that the law makes no distinctions and considers everything on the same level could affect the rate of conscientious objectors. Another fundamental problem is that the Italian legislation gives great importance to the time limit, but not to the reasons that would lead the mother to make this decision: the entire burden of treatment is therefore on the gynecologists and psychiatrists [9]. Although there are several studies on the ethical dilemmas raised in relation to TOP and on healthcare professionals’ opinions about the management, the literature that explores the experiences or the difficulties that operators encounter in clinical, moral and emotional manner is scarce [10]. For these reasons, the objectives of this study are to analyze the following: if the operators would change their status as objectors depending on the clinical situation and whether the pregnancy is in the first or second trimester; if the establishment of a multi-collegiate commission could influence the choice of being objector; and if the introduction of pharmacological rather than surgical methodologies can influence the operator’s choice.

## 2. Materials and Methods

This is an observational, transversal, descriptive study that involved a cohort made up of healthcare workers in Italy who participated in a web-survey.

The main inclusion criterion was being a health worker in the fields of Gynecology and Obstetrics or Anesthesiology. These medical categories are the most involved in TOP clinical procedures. The exclusion criteria were the lack of consent, and to not have been involved in TOP procedures for work. Operators were recruited by inviting them to participate in an anonymous online questionnaire distributed in Italy via social networks (i.e., Instagram, Inc., San Francisco, CA, USA, Android: 221.0.0.16.118 iOS), scientific societies (i.e., University of Piemonte Orientale), and messaging platforms (i.e., WhatsApp, Inc., Menlo Park, CA, USA 2.24.23.79). A link to the questionnaire was distributed through these channels. The responses were automatically organized anonymously on a Google data processing platform. The recruitment period started from the date of online dissemination of the interview in December 2021. In February 2022, the minimum sample size was reached, and we decided to evaluate these preliminary data as part of an ongoing research on other aspects regarding healthcare workers. Study design and data collection methods adhered to the STROBE guidelines [11]. This study was conducted in accordance with the ethical principles of the Declaration of Helsinki and was approved by the Ethics Committee of the AOU Maggiore of Novara, EC protocol number 286/21. Given the absence of validated questionnaires on the topic, the survey was developed de novo by a team of researchers (Supplementary Material) based on a model of similar questionnaires found widespread in the literature [12]. The questionnaire used was approved by the commission of the hospital and university ethics committee, made up of doctors and bioethicists due to the clinical and ethical aspects of the topic. It was decided to address participants with an online survey to expand the distribution and encourage participation. The questionnaire items are specifically aimed at detecting the socio-work characteristics and opinions regarding various situations that may be associated with TOP (Supplementary Material).

### Statistical Analysis

**Figure 1:**
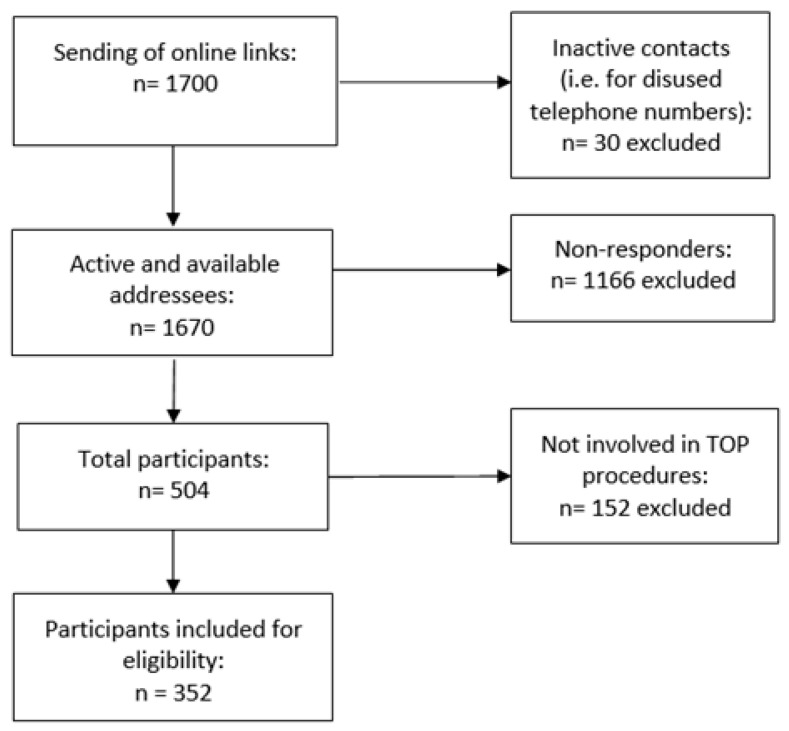
Recruited sample. TOP = Termination of pregnancy.

Based on the information from previous similar web-based studies, a response rate of 30.0% was expected [12]. Evaluating a 95% confidence interval (two-tailed) and a margin of error equal to 5.0%, we considered a minimum sample size of 350 patients (Figure 1). Data were analyzed at the completement of the enrollment. Results are presented in aggregate form, and it was not possible to derive information or make comparisons at an individual level. Analyses were performed to describe the various characteristics under consideration. Evaluations were summarized using descriptive statistics, absolute frequencies, and percentages.

## 3. Results

**Figure 2:**
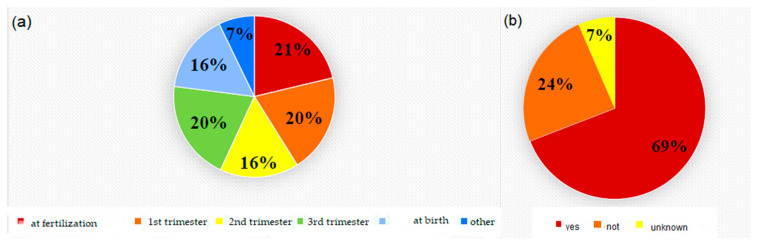
Answers about (**a**) the moment in which the embryo/fetus becomes a human being and (**b**) the suspension of conscientious objection in the case of first-trimester abortion secondary to sexual violence.

A total of 352 healthcare workers were included in this study; 88.6% were women and 11.4% were men. Only 20.8% declared to be objectors to voluntary abortion versus 79.2% of non-objectors. The job roles occupied were 36.5% nurses, 20.3% midwives, 18.0% gynecologists, 10.0% ‘other’, 8.9% anesthetists, 3.7% surgery room nurses, and 2.6% para-healthcare workers. The years of their work experience averaged mainly at 5 years (63.4%). For each work category, the majority declared to be non-objectors, without a specific prevalence in the different categories (Table 1). The age group that participated most in the survey was ≤to 35 years (79.3%), followed by ≤to 45 years (14.8%) and finally the group aged ≤to 55 (5.7%) (Table 2). A total of 85.8% of the participants were in a relationship, 69.9% had no children, 15.9% had one child, 11.9% had two children, and 2.3% had more than 2 children. 71.5% of the total sample worked at public hospitals, while the remaining 28.5% worked at other types of institutions (territorial services, private centers etc.). 59.5% of the participants did not have religious interest, while 40.0% defined themselves as religious believers, but 41.2% of these participants were not practicing (Table 1). Of the 20.8% of operators who declared themselves objectors, 80.8% objected for moral reasons, 48.7% for religious reasons, 12.8% for personal comfort and the remaining 14.1% declared for “other reasons” (more than an answer was admitted). In regard to the question ‘have you ever had personal experiences with voluntary termination of pregnancy?’, 84.5% did not. Regarding job experiences with TOP, 64.4% had direct interactions. Of the 349 operators who answered the question ‘have you ever had personal experience regarding voluntary termination of pregnancy?’, 295 operators declared that they had not; and of these, only 20.3% declared themselves to be conscientious objectors. Among those who declared to have had personal experiences in this regard (54 operators), 22.2% declared to be conscientious objectors. The participants were then asked what, in their opinion, is the moment in which the embryo/fetus becomes a human being; the answers obtained are very different: 21.0% answered at fertilization, 20.0% at first trimester, 20.0% at the third trimester, 16.0% at the second trimester, 16.0% at birth, and 7.0% answered ‘other’ (Figure 2). Those who identified themselves as objectors were asked whether they would suspend the objection in the case of only pharmacological abortion (within the first 90 days); 72.2% of operators answered they would not, while the remaining 27.8% affirmed that they would suspend the objection. The other question posed to objectors was whether the operator would suspend conscientious objection in the case of first-trimester abortion secondary to sexual violence, to which 69.0% agreed, 24.0% did not agree and 7.0% did not know (Figure 2).

The next question asked whether the operator would suspend the objection for an abortion in case of collegial decision, and 63.3% answered “yes” while 36.7% answered they would not. When asked whether the operator would suspend the objection in the event of TOP in the second trimester, 20.3% affirmed that they would suspend it while 79.7% stated they would not suspend. Another question was about whether the operator would suspend conscientious objection in the event of TOP following a serious maternal illness; from a total of 79 respondents, 72.2% answered “yes” and 27.8% answered “no”. The next question asked whether the operator would suspend conscientious objection in the event of TOP due to serious fetal disease (e.g., malformation not compatible with post-natal survival, genetic syndrome with unfavorable prognosis, etc.): 65.4% responded positively while 34.6% answered negatively. The last two questions were aimed at all participants and contained the main objective of the study: to evaluate whether the participation of a multidisciplinary panel could influence clinical practice and whether the abolition of the time limit can guarantee the patient a more serene choice. Of the 312 operators who responded, 45.8% declared that they would not change their attitude, 34.6% said that this would simplify clinical activity, 6.5% said that this would complicate daily practice, 11.3% did not want to express an opinion and 1.8% responded ‘other’. It emerged that of the non-objector operators who responded, among the main options, 27.7% declared that the introduction of a multidisciplinary commission could lighten the clinical practice; 46.7% stated that it would not change their activity, and 6.4% affirmed that it would complicate the clinical practice.

Among the objector operators who answered, 42.7% would lighten their clinical practice with a multidisciplinary commission, 17.8% declared that they would not change it, while 8.21% did not want to express an opinion. Finally, the last question was addressed to all participants, objectors and non-objectors, and it is about the abolition of the time limit of 22-gestational weeks to guarantee a more peaceful choice. It emerged that of the 343 healthcare professionals who responded to the question, 72.0% declared that the abolition can guarantee more time for the woman to reflect on the best choice to make, 12.5% said that it only increases the latency time, 6.4% said that it has no effect on the woman’s choice, 8.2% did not want to express an opinion, and 0.9% replied ‘other’.

## 4. Discussion

The main participation of female operators in the questionnaire correlates with the greater presence of women in the healthcare world [13]. Most of the participants declared that they were not conscientious objectors, contrary to what is seen in real life and what has been described in the literature in previous generations, i.e., 70.0% of Italian gynecologists declare themselves objectors [3,14]. Probably, in our case, there is a low percentage of conscientious objectors (20.8%) because the questionnaire was carried out mostly by young people (≤35 years). Younger operators are more interested in the topic, according to a recent survey conducted by the Italian Ministry of Health on a cohort of 21,217 people aged 18–49 [15]. The reason for the sample’s age could be online distribution. On the contrary, maybe if the questionnaire had been carried out in person, it could have had a greater response even in the population of operators aged >35 years. Another possible explanation for the low percentage of objectors in our sample could be that young people have different moral and religious principles compared to older generations. Growing up in a constantly evolving context has influenced the consolidation of the moral values of our modern society. Furthermore, religion seems to be practiced differently respect the past, and among the new generations there is a sort of distancing from religion. This can be one of the main reasons for the analyzed trend. As is known, religion and moral values have a strong impact on the percentage of objectors. For example, the Catholic Church considers the moment of conception as the beginning of human life and that an embryo is considered a person with the same legal rights [16,17]. In line with these concepts, from our results, many operators consider the embryo/fetus to be human from conception or, in any case, from the first trimester of pregnancy, maybe as a cultural consequence.

The intention to procreate is also decreasing compared to the past. Reasons could be mainly linked to economic and work factors, as the absence of social support for families with children, followed by reasons related to couple life or the personal sphere; finally, there could be other reasons, such as health problems or critical issues of family management. These are only some of the possible factors for turning to TOP [15].

Regarding the personal experiences of health workers, we can hypothesize that those who have never had experience in this regard more frequently declare themselves to be non-objectors. Another hypothesis could instead be that they do not declare themselves conscientious objectors because they are conditioned by the experience they have had. Operators with this kind of personal experience could also be more emotionally involved [18]. Personal experience could play a fundamental role in the choice of being an object or not. Assisting TOP is often defined as a stressful situation. This experience seems to be accompanied by recurring difficulties, such as the complex management of TOP in advanced gestational ages, the birth of live fetuses, or interruptions not related to pathologies [19]. Most midwives also affirmed that general clinical care appears to be suboptimal [20]. The emotional individual component of gynecologists and midwives is greatly underlined in the literature. There are few studies about the opinions of other types of healthcare professionals; this is a strong point of our study [21,22,23,24,25]. There is a strong need to analyze more and more the factors linked to the operators and the choice of being conscientious objectors.

In France, there is the possibility of TOP beyond the 22 gestational weeks in cases of serious maternal or fetal conditions, and the choice is not up to the patient, but to a multi-collegiate commission made up of specialists [26]. In our survey, the introduction of a multi-college commission would not change the clinical practice of non-objector operators. Furthermore, half of the objectors declared that they would lighten a collaborative decision for their clinical practice. Except in the case of personal choice for ethical/religious reasons, probably, operators would feel ‘exonerated’ from the weight of individual decisions with the inclusion of a multidisciplinary panel. With the introduction of a commission, the numeric problem of the healthcare operator could be partially solved. Another important concept that emerged from our web-survey is that health workers would not change their objector status by making a distinction between pharmacological and surgical abortion. Instead, although with a strong ethical impact, responses regarding sexual violence and maternal/fetal pathologies were oriented towards supporting the woman in choosing voluntary TOP, and these were also the answers from objector participants. Gender violence versus women is still a very hot social topic [27,28]. Most of the answers was in favor of abolishing the 22-week time limit to allow the woman more time to decide. In fact, fetal malformations are not always identified within 22 gestational weeks [29,30], so it may take longer to analyze the clinical situation and decide what should be done. Considering that in some regions several days are often required to find a facility for TOP procedures, many women can also face economic or social difficulties when requesting earlier diagnostic tests for the fetus [31].

However, considering the complex ethical discussion on life from its beginning, it is also essential to evaluate the protection of women’s well-being. For these reasons, it could be necessary to involve medical staff in the evaluation of TOP legislative applicability and in the periconceptional choice; it would be optimal to integrate their opinion right from the training during their university career, to be more conscious and to globally help women, thereby beginning the process of avoiding unwanted pregnancies till an eventual TOP [32,33].

The European situation is also very complex; for example, Ireland, Malta and Poland have restrictive laws. Luxembourg allows TOP on physical and mental health indications; Cyprus, Finland, and the UK include socio-economic indications. In all other European states, TOP can be performed in early pregnancy if requested. A total of 10.3 terminations were reported per 1000 women aged 15–49 years in Europe in 2008. Northern Europe (10.9/1000) and Central and Eastern Europe (10.8/1000) had higher rates than Southern Europe (8.9/1000) [34,35].

Our survey could serve as a reflection on the impact of TOP among healthcare workers to help determine some decisive aspects in the operators’ choice.

The main limitations of this study are related to the lower participation of workers over 35 years of age and of male sex operators, which is linked to the current demographic component of these professional classes. Our strengths are the statistical validity of the sampling number (to implement in future studies), the stratification on multiple healthcare figures, and the wide analysis from the clinical management to the personal attitude of abortion.

## 5. Conclusions

Most of the participants declared that the abolition of the time limit could have beneficial effects on the choice of patients. Furthermore, the operators confirmed that they would not change to be objectors regardless of the type of abortive method and the gestational age. The objectors in our sample affirmed that, in the event of voluntary abortion after experiencing sexual violence, maternal/fetal illness and/or with the introduction of a multi-collegiate commission, they would be willing to change their status.

## Figures and Tables

**Table 1:** Working roles of our sample and the correlation between objector status and religiosity.

Working Role	Not- Conscientious Objectors	Conscientious Objectors	Not-Religious Believers	Religious believers	Tot.*
**Nurses**	100 (79.4%)	26 (20.6%)	77 (61.0%)	49 (39.0%)	126
**Midwives**	57 (80.3%)	14 (19.7%)	41 (58.0%)	30 (42.0%)	71
**Gynecologists**	49 (77.8%)	14 (22.2%)	39 (62.0%)	24 (38.0%)	63
**Anesthetists**	26 (83.9%)	5 (16.1%)	21 (68.0%)	10 (32.0%)	31
**Surgery Room Nurses**	11 (84.6%)	2 (15.4%)	8 (61.5%)	5 (38.5%)	13
**Para-Health Workers**	6 (60.0%)	4 (40.0%)	4 (40.0%)	6 (60.0%)	10
**Other**	27 (77.1%)	8 (22.9%)	18 (51.0%)	17 (49.0%)	35

* Three operators did not answer the question about their working roles.

**Table 2:** Correlations among age, objector status, and religiosity of our sample.

AGE	Not- Conscientious Objectors	Conscientious Objectors	Not-Religious * Believers	Religious Believers
**≥35**	235 (84.2%)	44 (15.8%)	175 (62.9%)	103 (37.1%)
**≥45**	32 (61.5%)	20 (38.5%)	22 (42.3%)	30 (57.7%)
**≥55**	12 (60.0%)	8 (40.0%)	11 (55.0%)	9 (45.0%)
**≥65**	1 (100.0%)	-	1 (100.0%)	-

* One operator did not answer the question about religiosity.

## Data Availability

Data will be made available on request.

## Supplementary Materials

The following supporting information can be downloaded at: https://www.mdpi.com/article/10.3390/medicina60121984/s1, Questionnaire.
