# Supplementary material for: “To Be or Not to Be” a Conscientious Objector to Voluntary Abortion: An Italian Web-Survey of Healthcare Workers
Source: Medicina (Kaunas). 2024 Dec 2;60(12):1984. doi: 10.3390/medicina60121984 (PMC11728248; doi:10.3390/medicina60121984)
Supplement: Supplementary file 1 [file medicina-60-01984-s001.zip › medicina-3263206-supplementary.pdf]

- SUPPLEMENTAL MATERIAL-

WEB SURVEY

Our questionnaire is aimed at healthcare workers and aims to evaluate this issue globally:

"Would you change your opinion if the Italian law 194/78 were modified, with the possibility of expressing or not expressing your objection in particular conditions and/or in the context of a collegial multi-specialist choice?"

Currently in Italy a patient who does not wish to continue the pregnancy for various reasons (health, economic, social, family, work, etc...) can request voluntary termination of pregnancy, free of charge, within the first 90 days according to Law 194/78. From August 2020, for patients within the maximum limit of the 63rd day (9 weeks calculated starting from the first day of the last menstruation) it is possible to undergo pharmacological interruption with RU486.

According to the law 194/78, the voluntary interruption of pregnancy, after the first 90 days but within the 180th day, can be practiced when the pregnancy or birth involves a danger to the woman's life or when confirmed pathological processes, including those relating to significant anomalies or malformations of the fetus, which cause a danger to the physical or mental health of the woman. The pathological processes that constitute the cases envisaged in the previous article are ascertained by a doctor from the obstetric service, who can make use of the collaboration of specialists. Abroad, the various regulations on the interruption of the second trimester do not express a maximum limit of achievable gestational age and in many cases, as in France for example, require the meeting of a multidisciplinary commission (gynecologist, paediatrician surgeon, geneticist, bioethicist, etc.) who discusses the individual case and expresses a favorable opinion or not.

1) Age:

☐ ≤35 ☐ ≤45 ☐≤55 ☐≤65

2) Sex:

☐M ☐F ☐Other

3) Children:

☐0 ☐1 ☐2 ☐ >2

4) Partner (last 6 months):

☐Yes ☐No

5) Healthcare Role:

☐Gynecologist

☐Anesthetist

☐Nurse

☐Operating room nurse

☐Midwife

☐Para-health worker

☐Other

6) Years of experience:

☐≤5 years ☐ ≤10 years ☐≤20 years ☐other

7) Do you work in a hospital?

☐Yes ☐No

8) Would you define yourself as a practicing believer of any religion?

☐Yes ☐No ☐No answer

If yes, how many times a month do you go to your place of faith?

☐0 ☐ 1 ☐2 ☐3 ☐>3

9) Have you ever had personal experiences regarding a termination of pregnancy?

☐Yes ☐No ☐No answer

10) Have you ever had work experience regarding an interruption of pregnancy?

☐Yes ☐No ☐No answer

11) In your opinion, at what gestational age does an embryo/fetus become a human being?

☐At fertilization ☐1st trimester ☐2nd trimester ☐3rd trimester ☐at birth ☐ other

12) Would you define yourself as a conscientious objector?

☐Yes ☐No ☐No answer

13) If yes, choose from the following reasons (even more than one):

☐Religious ☐Moral ☐Practical/convenience ☐Legal risk ☐Other

14) If you answered Yes to question 12, would you suspend the objection for a pharmacological termination of pregnancy in the first trimester only?

☐Yes ☐No ☐No answer

15) If you answered Yes to question 12, would you suspend the objection for termination of pregnancy in the first trimester in the event of a pregnancy arising after sexual violence?

☐Yes ☐No ☐No answer

16) If you answered Yes to question 12, would you suspend the objection for termination of pregnancy only in the second trimester?

☐Yes ☐No ☐No answer

17) If you answered Yes to question 12, would you suspend the objection for second trimester pregnancy termination decided by a collegial opinion of specialists?

☐Yes ☐No ☐No answer

18) If you answered Yes to question 12, would you suspend the objection to termination of pregnancy in the event of serious maternal illness?

☐Yes ☐No ☐No answer

19) If you answered Yes to question 12, would you suspend the objection for termination of pregnancy in case of serious fetal disease (malformation not compatible with postnatal survival, genetic syndrome with unfavorable prognosis, etc.)?

☐Yes ☐No ☐No answer

20) A possible modification of the law in the interruption of the second trimester with the involvement of a multidisciplinary panel:

☐would not change clinical practice

☐would lighten the clinical practice (e.g. less sense of personal responsibility regarding patient's choice)

☐would complicate clinical practice

☐I don't want to express an opinion

21) Whether you are a non-objector or not, do you think that the abolition of a time limit within the law, at least in the case of interruption of the second trimester:

☐Can guarantee the patient a more serene choice with more time to decide, ask for a second opinion, wait for the results of genetic investigations, etc...

☐Has no effect on the choice to stop

☐Only increases the latency time without having any effect on the patient's choice

☐ I don't want to express an opinion
